# Community‐Acquired Pneumococcal Ventriculitis as a Complication of Otogenic Pneumococcal Meningitis: A Case Report

**DOI:** 10.1155/crdi/8876814

**Published:** 2026-07-14

**Authors:** Marco Seneghini, Jonas Deppe, Alexis P. R. Terrapon, Philipp Balcerak, Georg Kägi, Werner C. Albrich

**Affiliations:** ^1^ Division of Infectious Diseases, Infection Prevention and Travel Medicine, Cantonal Hospital St. Gallen, HOCH Health Ostschweiz, St. Gallen, Switzerland, kssg.ch; ^2^ Division of Neurology, Cantonal Hospital St. Gallen, HOCH Health Ostschweiz, St. Gallen, Switzerland, kssg.ch; ^3^ Division of Neurosurgery and Spine Center of Eastern Switzerland, Cantonal Hospital St. Gallen, HOCH Health Ostschweiz, St. Gallen, Switzerland, kssg.ch

**Keywords:** case report, ependymitis, otogenic meningitis, pneumococcal vaccination, pneumococcus, pyogenic ventriculitis, Serotype 3 *Streptococcus pneumoniae*, ventricular empyema, ventriculitis

## Abstract

Pyogenic ventriculitis is a rare and potentially fatal infection of the ventricular system. While most reported cases are nosocomial and catheter‐associated, ventriculitis as a complication of community‐acquired bacterial meningitis is uncommon, particularly in adults, and carries a poor prognosis. We report the case of an 82‐year‐old previously healthy and unvaccinated woman who presented with a one‐day history of fever, confusion, and purulent left‐sided otorrhea on a 2‐week background of flu‐like symptoms. Cranial computed tomography was consistent with otogenic meningitis complicated by pneumocephalus, and lumbar puncture confirmed purulent meningitis. Empirical therapy for meningitis with ceftriaxone, amoxicillin, metronidazole, and dexamethasone was initiated, and surgical source control of the otogenic focus was performed. Both blood and cerebrospinal fluid cultures grew *Streptococcus pneumoniae* with a penicillin minimum inhibitory concentration of < 0.03 mg/L. Serotyping by multiplex PCR with confirmatory Quellung reaction, performed at the Swiss National Reference Center for Invasive Pneumococci, identified Serotype 3. Despite penicillin G monotherapy, fever and impaired consciousness persisted, and repeat imaging revealed meningitis‐associated pyogenic ventriculitis with intraventricular debris. Adjunctive rifampicin and a series of cerebrospinal fluid drainage interventions (lumbar drain followed by external ventricular drain) failed to substantially improve neurological outcome. Antibiotic therapy was continued for 9 weeks, after which the patient was transferred to long‐term institutional care with severe residual neurological impairment. This case illustrates the diagnostic and therapeutic challenges of meningitis‐associated pyogenic ventriculitis in adults and the disproportionate severity of disease caused by Serotype 3 *S. pneumoniae*, whose unusually thick mucoid capsule promotes immune evasion and the formation of viscous purulent debris that hampers source control. The case is consistent with Swiss surveillance data showing that Serotype 3 remains a leading cause of invasive pneumococcal disease in older adults and serves as a sentinel event highlighting persistent gaps in adult pneumococcal immunization.

## 1. Introduction

Pyogenic ventriculitis, also known as ventricular empyema, ependymitis, or pyocephalus, is a rare and potentially fatal infection of the ventricular system which can lead to severe neurological sequelae in survivors [1]. There is no universally accepted definition [1, 2]. The majority of reported cases in the medical literature occurred in association with ventricular catheters [3]. In contrast, nonsurgery‐related primary bacterial ventriculitis or ventriculitis as a complication of meningitis is rarely described [4]. Here, we report the case of an 82‐year‐old female with pyogenic ventriculitis presenting as a complication of otogenic meningitis caused by a Serotype 3 *Streptococcus pneumoniae*.

## 2. Case Presentation

An 82‐year‐old woman with no significant past medical history and without previous pneumococcal vaccination initially presented at a local emergency department with a one‐day history of fever and confusion. She had been experiencing flu‐like symptoms for 2 weeks and ear pain of 1‐day duration prior to admission. There was no history of head trauma. Upon presentation, she was febrile (38.7°C), hypertensive (165/99 mmHg), and tachycardic (120/min). She had a severely reduced conscious state (GCS 9, E2V2M5) with round, equal, and reactive pupils. Clinical examination revealed meningism and purulent left‐sided otorrhea. A full blood count showed leukocytosis of 19,000/mm^3^ with neutrophilia (84.7%). Serum CRP level was high (206 mg/L), and procalcitonin was not elevated (0.34 μg/L) (Table 1).

**TABLE 1:** Laboratory findings from admission (d1) to 5 days after switching from penicillin G to ceftriaxone (d48).

	d1	d6	d13	d21	d33	d48
*Blood analysis*						
CRP (< 5 mg/L)	206	73	18	57	56	6
Procalcitonin (< 0.5 μg/L)	0.34			0.06		
Leukocytes (4.0–10.0 G/L)	32.0	28.3	21.0	13.0	11.6	13.9

*CSF analysis*						
Cell count (< 3/μL)	7127		1561	224		
Total protein (0.15–0.45 g/L)	10.5		5.0	2.6		
Albumin (0.06–0.24 g/L)	6.0		3.0			
Lactate (< 2.4 mmol/L)	21		7.0	8.5		
Glucose (2.2–3.9 mmol/L)	< 0.6		3.0	2.0		

*Microbiology*						
Blood culture	positive		negative	negative		
CSF culture	positive		negative	negative		

*Note:* CSF = cerebrospinal fluid; d = day.

A cranial computer tomography (CT) scan with contrast demonstrated wall thickening of all paranasal sinuses, subtotal opacification of the left tympanic cavity and mastoid air cells, as well as gas inclusions in the internal acoustic meatus, petroclival space, suprasellar cistern, and around the pons, consistent with otogenic meningitis complicated by pneumocephalus (Figure 1). There was no evidence of fractures or other traumatic lesions. A chest CT scan appeared normal without pulmonary infiltrates.

**FIGURE 1:**
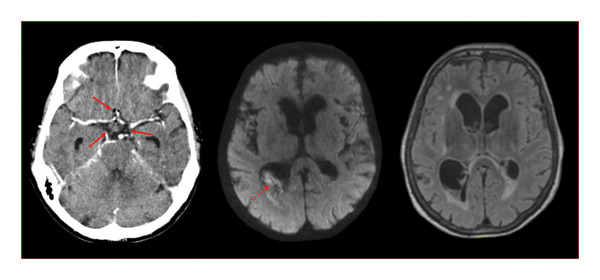
Axial brain CT and MRI scans of the patient from admission (left) to discharge (right). Visible gas inclusions (arrows) next to the cerebral arterial circle of Willis in a contrast CT scan on the day of hospital admission (left). DWI scan on Day 12 showing ventriculitis (dotted arrow, middle). FLAIR sequence 5 weeks after admission revealing partial regression of ventriculitis and dilatation of the ventricular system (right). CT = computed tomography, DWI = diffusion‐weighted imaging, FLAIR = fluid‐attenuated inversion recovery, and MRI = magnetic resonance imaging.

Lumbar puncture revealed turbid cerebrospinal fluid (CSF). Opening pressure was not measured. CSF analysis yielded granulocytic pleocytosis, lactate elevation, nondetectable glucose, as well as signs of blood–brain barrier disruption with increased protein and albumin levels (Table 1). After collection of blood cultures, the patient was treated empirically for meningitis with ceftriaxone (2 g q12 h), amoxicillin (2 g q4 h), metronidazole (500 mg q6 h), and dexamethasone (10 mg q6 h). With suspected otogenic pneumococcal meningitis, the patient was referred to our tertiary care center for surgical management, including postauricular antrotomy, myringotomy, and insertion of a tympanic ventilation tube. Amoxicillin was stopped after CSF microscopy confirmed Gram‐positive diplococci. Both blood and CSF cultures grew *S. pneumoniae* Serotype 3 with a minimum inhibitory concentration (MIC) for penicillin < 0.03 mg/L. Serotyping was performed by multiplex PCR with confirmatory Quellung reaction at the Swiss National Reference Center for Invasive Pneumococci. The antibiotic regimen was then switched to penicillin G while dexamethasone was continued for 4 days. However, the treatment over the following days failed to resolve the fever and poor neurological status with reduced level of consciousness. Alternative sources of infection were excluded with repeat blood cultures, respiratory viral screening, and chest radiography. A repeat cranial CT scan revealed regression of pneumocephalus but new hyperdense debris in the occipital horns of the lateral ventricles consistent with pyogenic ventriculitis. Ciprofloxacin and hydrocortisone‐containing ear drops were added to the treatment regimen as otoscopy revealed persistent pus in the left ear canal. On Day 12 of hospitalization, repeat blood and CSF analyses demonstrated an overall decrease in inflammatory markers, but corresponding cultures remained negative (Table 1). A brain MRI scan indicated persistent ventriculitis with intraventricular pus and increasing ventricular dilation as a possible sign of beginning hydrocephalus (Figure 1). Given the lack of clinical improvement with unchanged lethargy and somnolence as well as radiological evidence of persistent ventriculitis, rifampicin (600 mg qd) was added on Day 13 after hospital transfer for improved bactericidal activity [5]. Rifampicin was discontinued after 9 days due to a possible drug‐induced fever and lack of marked neurologic improvement. Subsequently, the patient defervesced. Hydrocephalus was suspected as a cause of persistent impaired consciousness. Since there was no sign of CSF flow obstruction on MRI, a lumbar drain was placed in order to treat communicating hydrocephalus and enable for repeated CSF sampling. Due to suspicion of obstruction or dislocation and repeated unsuccessful attempts at replacing the lumbar drain, it was removed, and an external ventricular drain (EVD) was surgically inserted after 2 days. Due to a lack of neurological improvement despite adequate drainage volumes and correct intraventricular location of the EVD, it was in turn removed after 5 days. Five weeks after admission, the patient was still predominantly somnolent with only short phases of wakefulness where she was able to follow simple verbal commands, speak a few words, and sit upright with assistance. Nutrition was provided via nasogastric tube. Because of the impaired consciousness, an electroencephalogram was performed, which showed continuous generalized periodic discharges, correlating to severe brain damage, but without further features supporting epileptic seizures. A repeat cranial MRI scan revealed partial regression of ventriculitis, and the patient was transferred to a rehabilitation center. At that time, to facilitate antibiotic administration, treatment with penicillin G was replaced by ceftriaxone twice daily, 1 week before transfer to the rehabilitation center. After a total of 9 weeks, antibiotic therapy was stopped as both leukocyte count and CRP level were normal and repeat brain MRI demonstrated a significantly reduced meningeal and ventricular inflammation and a minimal residual pneumocephalus in the right lateral ventricular anterior horn (Figure 1). Clinically, the patient showed no improvement with persistent severely impaired consciousness during the following neurological rehabilitation and developed a spasticity of all limbs, pronounced on the left. A repeated brain MR scan revealed, once again, possible signs of hydrocephalus. Therefore, a CSF tap test was performed without relevant clinical improvement. A percutaneous gastrostomy tube was placed for nutrition, and the patient remains institutionalized with the need of care in all activities of daily living.

A timeline of clinical events, investigations, and key therapeutic interventions from admission to long‐term follow‐up is summarized in Table 2.

**TABLE 2:** Timeline of main clinical events, investigations, and therapeutic interventions.

Day	Clinical event/investigation	Therapeutic intervention
Day 0 (admission)	Fever, confusion, GCS 9, purulent left otorrhea; CT consistent with otogenic meningitis and pneumocephalus; turbid CSF with granulocytic pleocytosis	Empirical IV ceftriaxone + amoxicillin + metronidazole + dexamethasone; blood and CSF cultures collected
Day 1‐2	Transfer to tertiary center; Gram stain: Gram‐positive diplococci	Postauricular antrotomy, myringotomy, tympanic ventilation tube; amoxicillin stopped
Day 3‐4	Blood and CSF cultures: *S. pneumoniae*, penicillin MIC < 0.03 mg/L; multiplex PCR + Quellung confirms Serotype 3	Switch to penicillin G; dexamethasone continued for 4 days
∼Day 6	Persistent fever, GCS unchanged; alternative sources excluded	Continued penicillin G
∼Day 10–12	Repeat CT/MRI: regression of pneumocephalus, new intraventricular debris, ventricular dilatation ‐ ventriculitis confirmed	Topical ciprofloxacin/hydrocortisone ear drops added
Day 13	No clinical improvement	Rifampicin 600 mg daily added
∼Day 22	Possible drug‐induced fever; no neurological improvement	Rifampicin stopped; subsequent defervescence; lumbar drain placed
∼Day 24	Suspected drain dysfunction	External ventricular drain (EVD) inserted surgically
∼Day 29	Adequate drainage but persistent impaired consciousness	EVD removed
Week 5	EEG: continuous generalized periodic discharges, no seizures; MRI: partial regression of ventriculitis	Switch from penicillin G to IV ceftriaxone twice daily; transfer to neurorehabilitation
Week 9	Normalized leukocytes and CRP; MRI: marked reduction of ventricular and meningeal inflammation, minimal residual pneumocephalus	Antibiotic therapy stopped (total 9 weeks)
Long‐term follow‐up	Persistent severe impaired consciousness, spasticity (left > right); CSF tap test without clinical benefit	Percutaneous gastrostomy; long‐term institutional care

## 3. Discussion

Ventriculitis is a serious condition that involves inflammation of the lining of the ventricles and ventricular fluid, which can lead to devastating long‐term neurological damage if not treated promptly and effectively. It is most commonly seen in patients after placement of ventricular catheters such as shunts or EVDs, in patients who have undergone other types of neurosurgery [6–9] or after trauma with (basilar) skull fractures [1, 10]. In order to prevent ventriculitis, various technologies have emerged, such as antibiotic‐impregnated and silver‐coated catheters [8]. Nosocomial ventriculitis is mostly caused by *Staphylococcus aureus*, coagulase‐negative staphylococci, or various Gram‐negative bacilli and *Cutibacterium acnes* [11, 12], whereas posttraumatic cases are mainly due to streptococci or Gram‐negative rods [1].

In contrast, nonsurgery‐related ventriculitis usually occurs as a complication of bacterial meningitis and is most commonly reported in infants [13–15]. Reports of such cases in adults are indeed very rarely reported in the medical literature, including one case of recurrent pyogenic ventriculitis [16–19].

The clinical manifestations of pyogenic ventriculitis can be heterogeneous, depending on the severity of the inflammation and the underlying cause [16], and it is not uncommon for patients to have an indolent presentation without signs of meningeal irritation [4]. Patients are frequently of advanced age. The most common causative organisms in primary ventriculitis are *Streptococcus pneumoniae, Neisseria meningitidis*, and *Staphylococcus aureus* [4]. In our case, involving an elderly but otherwise previously healthy woman, *S. pneumoniae* was identified in both blood and CSF cultures. Strictly speaking, given the clear otogenic source and the subsequent development of meningitis with ventricular extension, our case is best classified as meningitis‐associated (secondary) rather than primary pyogenic ventriculitis.

The diagnosis of ventriculitis typically involves a combination of imaging tests, as well as laboratory analysis of CSF and blood cultures. However, a universally accepted definition or diagnostic criteria is lacking [1, 2, 16]. Brain imaging, including CT and MRI, is crucial in the diagnostic process, the latter offering greater sensitivity compared to CT. One of the most characteristic radiologic features of pyogenic ventriculitis is the presence of ventricular debris on imaging [20]. Other typical radiologic features include dilated ventricles, hyperintense periventricular signal, and ependymal enhancement [20]. In our patient’s case, despite 12 days of hospitalization and appropriate antibiotic therapy, pneumocephalus and intraventricular pus persisted on repeated brain imaging, which may suggest an unfavorable prognosis.

According to a recent retrospective study including patients with ventriculitis of various etiologies (brain abscess, meningitis, intraventricular catheter‐related, postneurosurgery, and hematogenous spread), this pathology is associated with a mortality rate of 30% and neurological sequelae in 60% of survivors [21]. Poor prognostic factors included age > 65 years, GCS score < 13 on admission, status epilepticus, hydrocephalus, and positive CSF culture [21], all of which were present in our patient, with the exception of status epilepticus.

Serotype 3 pneumococcus is a particularly virulent pneumococcal serotype, known to cause more severe symptoms and a higher mortality rate compared to other strains of pneumococcus, being characterized by a fatality rate of 30%–47% [22].

A defining feature of Serotype 3 *S. pneumoniae* is its unusually thick mucoid polysaccharide capsule, which is significantly larger than that of most other pneumococcal serotypes. This capsule is shed in large quantities during infection, impairs complement deposition, and reduces opsonophagocytic killing, thereby facilitating immune evasion [22]. These features also promote the formation of highly viscous purulent debris, which may explain the radiological persistence of intraventricular debris and the difficulty in achieving adequate source control in our patient despite favorable penicillin MICs, the addition of rifampicin, and repeated CSF drainage interventions.

Despite mature pediatric pneumococcal conjugate vaccine (PCV) programs, Swiss national surveillance data covering 2012–2022 have shown that Serotype 3 remains the leading cause of invasive pneumococcal disease (IPD) in adults aged ≥ 65 years, with a substantial residual case‐fatality rate [23]. Our case is consistent with this epidemiological pattern and underlines the persistent public‐health relevance of Serotype 3 in older adults in Switzerland.

This case can be regarded as a sentinel event highlighting the gap in adult pneumococcal immunization. Our patient was 82 years old, previously healthy, and had never received a pneumococcal vaccine. Serotype 3 is included in PCV13, PCV15, PCV20, PCV21, and PPSV23. However, Serotype 3 has long been considered the “hole in coverage” of PCVs, as antibody responses to its polysaccharide are lower and functionally less effective than for other vaccine serotypes [24] Newer higher‐valent conjugate vaccines (PCV15 and PCV21) elicit improved immunogenicity against Serotype 3, but the clinical implications of this enhanced response on Serotype 3 IPD remain to be determined and will need to be assessed in long‐term cohorts [25]. Even if our patient had been immunized prior to disease onset, vaccination might therefore not have reliably prevented her from this disease. Nevertheless, structured pneumococcal vaccination should be considered for survivors of severe IPD during their transition to rehabilitation, once acute inflammation has resolved, and immunosuppressive therapy has been discontinued, alongside other age‐appropriate vaccinations (e.g., influenza and SARS‐CoV‐2).

All the cited factors likely contributed to the poor neurological outcome in our patient, even though targeted antibiotic therapy was initiated promptly after admission and continued for over 9 weeks. Overall, the optimal duration of antibiotic therapy for primary ventriculitis remains unclear due to the limited number of studies and their heterogeneity. Some studies on drain‐associated ventriculitis and meningitis suggested shorter durations (10–14 days) [12], whereas a longer durations of antibiotic therapy, similar to that used for brain abscesses (6–12 weeks) [26], have been recommended for primary ventriculitis, especially in cases where patients exhibit significant comorbidities, persistent positive CSF cultures, delayed clinical improvement, or other high‐risk factors [16, 27]. This extended treatment duration aims to ensure complete eradication of the infection and reduce the risk of relapse.

In summary, ventriculitis is a serious and potentially life‐threatening condition that requires timely diagnosis and treatment. Individuals at particular risk, such as patients with (history of) ventricular catheters, elderly, or immunocompromised, ought to be carefully monitored for signs of infection and provided with necessary medical care if required. The increased availability of advanced brain imaging techniques, such as MRI, has undoubtedly contributed to the rise in diagnoses and is expected to further increase their frequency in the near future.

With this case report, we aim to share our personal experience and raise awareness about a neurological entity which is still relatively underrecognized but carries a potentially devastating clinical impact. Further studies are required to accurately characterize the epidemiology and define the optimal therapeutic regimen of primary pyogenic ventriculitis.

## 4. Conclusion

Ventriculitis is a serious and potentially life‐threatening condition that requires timely diagnosis and treatment. Individuals at particular risk, such as patients with (history of) ventricular catheters, elderly, or immunocompromised, ought to be carefully monitored for signs of infection and provided with necessary medical care if required. The increased availability of advanced brain imaging techniques, such as MRI, has undoubtedly contributed to the rise in diagnoses and is expected to further increase their frequency in the near future.

With this case report, we aim to share our personal experience and raise awareness about a neurological entity which is still relatively underrecognized but carries a potentially devastating clinical impact. We also wish to underline that *S. pneumoniae* Serotype 3 continues to cause severe invasive disease in older adults, with pathophysiological features that translate into clinically relevant difficulties in achieving source control, and that every case of severe IPD in an unvaccinated older adult should be regarded as a sentinel event prompting structured pneumococcal vaccination of the survivor. Further studies are required to accurately characterize the epidemiology and define the optimal therapeutic regimen of pyogenic ventriculitis.

## Author Contributions

Marco Seneghini and Jonas Deppe were directly involved in the clinical care of the patient, conceived the case report, performed the literature review, and drafted the manuscript. Alexis P. R. Terrapon was involved in the neurosurgical management of the patient and contributed to the neurosurgical content of the manuscript. Philipp Balcerak and Georg Kägi were responsible for the neurological assessment and follow‐up of the patient and critically revised the neurological content. Werner C. Albrich supervised the infectious diseases management of the patient, contributed epidemiological and pathophysiological content, and critically revised the manuscript for important intellectual content.

## Funding

No funding or sponsorship was received for this study or the publication of this article.

## Disclosure

Part of this work was previously presented as a poster entitled “Severe community‐acquired pneumococcal ventriculitis as a complication of otogenic pneumococcal meningitis –a case report” at the Joint Annual Meeting 2023 of the Swiss Society for Infectious Diseases (SSI), held September 13–15, 2023, in Zürich, Switzerland. All authors read and approved the final version of the manuscript. This case report has been prepared in accordance with the CARE (CAse REport) guidelines.

## Ethics Statement

In accordance with Swiss federal law on research involving human subjects (Human Research Act, HRA), single anonymized case reports do not require formal review by a Cantonal Ethics Committee. The patient’s legal representative provided written informed consent for publication of the clinical history, laboratory data, and anonymized radiological images. A copy of the signed consent form is available from the corresponding author on request. The consent form is available for review by the Editor‐in‐Chief on request.

## Consent

Please see the Ethics Statement.

## Conflicts of Interest

The authors declare no conflicts of interest. WCA has received travel grants (Pfizer, Gilead) and reimbursement for advisory boards (Pfizer, MSD, Sanofi‐Aventis, GSK, OM Pharma, Janssen, and Moderna) or presentations (Pfizer, MSD, GSK, and Gilead), paid to his institution.

## Supporting Information

Additional supporting information can be found online in the Supporting Information section.

## Supporting information


**Supporting Information** CARE checklist.

## Data Availability

All data generated or analyzed during this case report are included in this published article. Additional anonymized data supporting the findings are available from the corresponding author on reasonable request, subject to applicable Swiss data‐protection regulations.
