# Supplementary material for: Community‐Acquired Pneumococcal Ventriculitis as a Complication of Otogenic Pneumococcal Meningitis: A Case Report
Source: Case Rep Infect Dis. 2026 Jul 14;2026:8876814. doi: 10.1155/crdi/8876814 (PMC13366205; doi:10.1155/crdi/8876814)
Supplement: Supplementary file 1 — Supporting Information CARE checklist. [file CRDI-2026-8876814-s001.docx]

**CARE (CAse REport) checklist**

**Manuscript:** Community-acquired pneumococcal ventriculitis as a complication of otogenic pneumococcal meningitis – a case report.

**Authors**: Marco Seneghini, Jonas Deppe, Alexis P. R. Terrapon, Philipp Balcerak, Georg Kägi, Werner C. Albrich

| **Topic** | **Item No.** | **Checklist item description** | **Location in manuscript** |
| --- | --- | --- | --- |
| **Title** | 1 | The words "case report" should be in the title along with the diagnosis or intervention of primary focus. | Title page |
| **Key Words** | 2 | 2 to 5 key words that identify topics in this case report. | Title page (8 keywords, including "case report") |
| **Abstract** | 3a | Introduction – What is unique about this case? What does it add to the medical literature? | Abstract, opening sentences |
|  | 3b | Main symptoms and/or important clinical findings. | Abstract |
|  | 3c | The main diagnoses, therapeutic interventions, and outcomes. | Abstract |
|  | 3d | Conclusion – What is(are) the main "take-away" lesson(s) from this case? | Abstract, closing sentences |
| **Introduction** | 4 | One or two paragraphs summarising why this case is unique with references. | Introduction |
| **Patient information** | 5a | De-identified patient specific information. | Case Presentation, ¶1 (no name, no exact dates) |
|  | 5b | Primary concerns and symptoms of the patient. | Case Presentation, ¶1 |
|  | 5c | Medical, family and psycho-social history including relevant genetic information. | Case Presentation, ¶1 |
|  | 5d | Relevant past interventions with outcomes. | N/A – no relevant prior interventions |
| **Clinical findings** | 6 | Significant physical examination and important clinical findings. | Case Presentation, ¶1; Table 1 |
| **Timeline** | 7 | Historical and current information organised as a timeline. | Table 2 |
| **Diagnostic assessment** | 8a | Diagnostic methods (examination, laboratory testing, imaging, surveys). | Case Presentation, ¶2–4; Table 1; Figure 1 |
|  | 8b | Diagnostic challenges. | Case Presentation, ¶3–4 (persistent inflammation; alternative sources excluded); Discussion ¶4 |
|  | 8c | Diagnostic reasoning including other diagnoses considered. | Case Presentation, ¶3–4 |
|  | 8d | Prognostic characteristics where applicable. | Discussion, ¶5 |
| **Therapeutic intervention** | 9a | Types of intervention (pharmacological, surgical, preventive, self-care). | Case Presentation, ¶3 |
|  | 9b | Administration of intervention (dosage, strength, duration). | Case Presentation, ¶3 (specific doses, intervals and durations) |
|  | 9c | Changes in intervention with rationale. | Case Presentation, ¶3 (de-escalation; switch to penicillin G; rifampicin added/stopped; CSF drainage) |
| **Follow-up and outcomes** | 10a | Clinician and patient assessed outcomes. | Case Presentation, ¶3; Conclusion |
|  | 10b | Important follow-up diagnostic and other test results. | Case Presentation, ¶3; Table 1; Figure 1 |
|  | 10c | Intervention adherence and tolerability. | Case Presentation, ¶3 (inpatient parenteral therapy under direct observation) |
|  | 10d | Adverse and unanticipated events. | Case Presentation, ¶3 (possible rifampicin-induced fever; lumbar drain dysfunction) |
| **Discussion** | 11a | Scientific discussion of strengths AND limitations. | Discussion (lack of universal diagnostic criteria; limited evidence on antibiotic duration) |
|  | 11b | Discussion of the relevant medical literature with references. | Discussion (27 references) |
|  | 11c | Scientific rationale for any conclusions. | Discussion (serotype 3 pathophysiology; mapping of prognostic factors) |
|  | 11d | Primary "take-away" lessons. | Conclusion |
| **Patient perspective** | 12 | The patient should share their perspective on the treatment(s) received. | N/A – patient has severe persistent impaired consciousness; consent obtained from legal representative |
| **Informed consent** | 13 | Did the patient give informed consent? Provide if requested. | Yes – written consent from legal representative (see Ethics Approval and Consent for Publication statements) |
